# Long-term Treatment With Metyrapone in a Man With Ectopic Cushing Syndrome

**DOI:** 10.1210/jcemcr/luac008

**Published:** 2022-11-29

**Authors:** Tin Wai Wong

**Affiliations:** Department of Medicine & Geriatrics, Ruttonjee & Tang Shiu Kin Hospitals, Wan Chai 999077, Hong Kong

**Keywords:** ectopic Cushing syndrome, thymic neuroendocrine tumor, metyrapone

## Abstract

This is a unique case of ectopic adrenocorticotropic hormone (ACTH)-secreting mediastinal neuroendocrine tumor, presumably thymic in origin, with suspected lung metastasis in a 61-year-old man who was successfully managed with long-term metyrapone alone. He presented with severe hypokalemia and hypertension, complicated with psychosis and vertebral collapse. He survived through a complicated course of pulseless ventricular tachycardia arrest and a severe Cushing storm due to drug nonadherence. For 3 years since diagnosis, he remained stable on metyrapone, and was able to achieve biochemical eucortisolism, with normalization of ACTH and cortisol levels. In addition, his tumor was reduced in size and the suspicious lung metastasis regressed.

Ectopic adrenocorticotropic hormone (ACTH) syndrome (EAS) represents 26% of ACTH-dependent Cushing syndrome (CS) [1]. Although rare, it often leads to rapid development of intense hypercortisolism, including electrolyte disturbances, uncontrolled hypertension, psychosis, cardiac events, and risk of opportunistic infection. Most primary endocrine tumors responsible for EAS are located in the chest, with bronchial carcinoids and small cell lung carcinoma being the most common causes, followed by thymic neuroendocrine tumor (NET). While surgery remains the optimal curative option, the efficacy of steroidogenesis inhibitors, chemotherapy, or radiotherapy has not been well established. This is a case of a presumably ACTH-secreting thymic NET (histological proof unavailable), which illustrates the fulminant features of EAS and demonstrates that long-term treatment with metyrapone could be safe and effective in biochemical and tumor control in the absence of surgery.

## Case Presentation

A 61-year-old gentleman first presented to emergency department for non-ST elevation myocardial infarction in February 2019. Coronary angiogram showed two-vessel disease, and percutaneous coronary intervention was performed. During hospitalization, he had persistent high blood pressure and hypokalemia. He was later discharged with 4 antihypertensive agents plus potassium chloride sustained release (slow K) 600 mg 3 times daily. Following the next 6 months, he developed progressive bilateral lower limb weakness, and slow K was increased to 1200 mg 3 times daily. He was readmitted in August 2019 with a potassium level of 2.0 mmol/L (mEq/L). In addition to proximal myopathy, he had developed low back pain and auditory and visual hallucination over the past 3 months.

On physical examination, he had a proximal lower limb power of grade 3/5. On closer inspection, he had cushingoid features with facial fullness and plethora, dorsocervical and supraclavicular fat pads, hyperpigmentation of sun-exposed regions, and mild central obesity.

Laboratory tests (Table 1) revealed severe hypokalemia, metabolic alkalosis, and a random cortisol level of 1172 nmol/L (42 mcg/dL). Hormonal analysis confirmed the diagnosis of an ACTH-dependent CS (Table 2). A 24-hour urine steroid profile detected a tremendous amount of free cortisol, metabolites of cortisol, and corticosterone, compatible with a picture of an extremely hyperactive adrenocortex with immature steroidogenesis due to intense ACTH stimulation. A 24-hour urine catecholamine level was normal.

**Table 1.** Biochemical results at presentation

Test	Result	Reference range
Sodium	149 mmol/L (mEq/L)	136-145 mmol/L (mEq/L)
Potassium	2.0 mmol/L (mEq/L)	3.4-5.0 mmol/L (mEq/L)
Urea	6.1 mmol/L (mEq/L)	2.7-7.2 mmol/L (mEq/L)
Creatinine	63 μmol/L (0.71 mg/dL)	69-110 μmol/L (0.78-1.24 mg/dL)
Bicarbonate	37.5 mmol/L (mEq/L)	20.0-26.0 mmol/L (mEq/L)
Base excess	14.2 mmol/L (mEq/L)	−3.0-3.0 mmol/L (mEq/L)
Blood pH	7.59	7.35-7.45
Glucose	5.1 mmol/L (92 mg/dL)	
HbA1c	4.9%	

**Table 2.** Hormone analysis at diagnosis

Test	Result	Reference range
Plasma ACTH	124 pg/mL (27 pmol/L)	≤ 46.0 pg/mL (≤ 10 pmol/L)
AM cortisol*^a^*	1659 nmol/L (60 mcg/dL)	185-624 nmol/L (6.7-22.6 mcg/dL)
Plasma renin assay	0.26 ng/mL/h	At rest 0.20-2.8 ng/mL/h
Aldosterone	< 50 pmol/L (< 1.8 ng/dL)	Standing 111-860 pmol/L (4-31 ng/dL) Recumbent 28-444 pmol/L (1-16 ng/dL)
24-hour urine free cortisol*^b^*	> 1700 nmoL/day (> 62 mcg/day)	22-157 nmoL/day (0.8-5.7 mcg/day)
AM cortisol after 1-mg overnight DST	1594 nmol/L (58 mcg/dL)	< 50 nmol/L (< 1.8 mcg/dL)
CRH stimulation test @ 30-45 min	11% cortisol increase from baseline 16% ACTH increase from baseline	

Abbreviations: ACTH, adrenocorticotropic hormone; CRH, corticotropin releasing hormone; DST, dexamethasone suppression test.

a Serum cortisol is measured using Beckman immunoassay method.

b Urine free cortisol is performed using liquid chromatography–tandem mass spectrometry.

**Figure 1.**
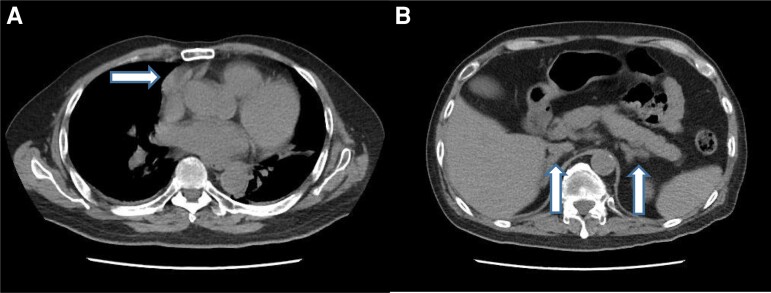
Computed tomography at presentation. A, ACTH-secreting right anterior mediastinal mass (arrow). B, bilateral adrenal hyperplasia (arrows).

**Figure 2.**
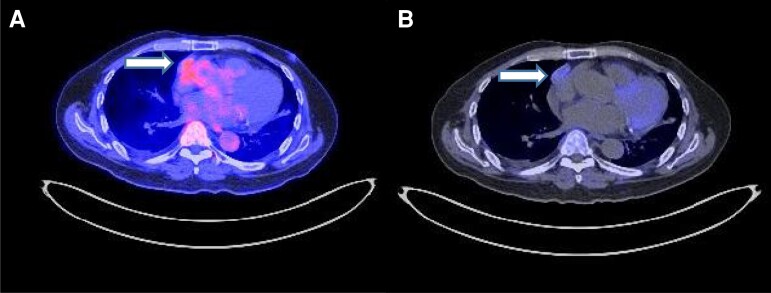
^18^FDG & ^68^Ga-DOTATATE positron emission tomography (PET)/CT at presentation demonstrating a right anterior mediastinal mass of 3.3 cm × 1.2 cm, with mild dual-tracer avidity. A, image with ^18^FDG tracer. B, image with ^68^Ga-DOTATATE tracer.

Magnetic resonance imaging (MRI) of the pituitary did not reveal a pituitary adenoma. Computed tomography (CT) of his thorax, abdomen, and pelvis revealed a smooth oval right anterior mediastinal mass measuring 3.6 cm × 1.6 cm × 2.6 cm (transverse [TS] × length [LS] × cranial-caudal [CC]), and bilateral diffusely thickened adrenals (Fig. 1A and 1B). Positron emission tomography (PET)/CT with fluorine-18-fluorodeoxyglucose (^18^FDG) and gallium-68-DOTATATE (^68^Ga-DOTATATE) demonstrated a mildly dual-tracer avid mediastinal mass, which had no invasion into the right atrium (Fig. 2A and 2B). There were also 2 right-side hypermetabolic lung opacities, measuring 1.8 cm × 0.9 cm and 1.5 cm × 1.2 cm with mildly increased ^68^Ga-DOTATATE activity and more intense ^18^FDG activity. There were multilevel partial vertebral collapses at T7 and T9-L5, which were not suggestive of bone metastases, but likely as a result of cortisol excess. Putting everything into context, the diagnosis was an ectopic ACTH-secreting mediastinal NET, with suspected lung metastasis.

## Treatment

He was treated with metyrapone 250 mg 3 times daily and hydrocortisone 10 mg twice daily as a block and replace regimen. Mean serum cortisol day-curve (CDC) was 200 nmol/L (7 mcg/dL) (normal range, 150-300 nmol/L; 5-11 mcg/dL). Resection of the mediastinal mass together with right lung wedge resection of the lung lesions was originally scheduled for October 2019. A few days before surgery, he developed cardiac arrest due to pulseless ventricular tachycardia. He was successfully resuscitated and had no neurological damage. A repeated coronary angiogram did not reveal significant stenosis accounting for the cardiac event. Repeated CT of the thorax in October 2019 found a significant reduction in size of the mediastinal mass measuring 2.3 cm × 1.2 cm × 1.7 cm (TS × LS × CC), and the right lung nodules showed marked interval decrease in size. Given a high cardiovascular risk at the time, and favorable treatment response, surgery was suspended. He underwent implantation of an implantable cardioverter defibrillator (ICD) in November 2019.

## Outcome and Follow-Up

Drug regimen was titrated to metyrapone 250 mg 3 times daily with hydrocortisone stopped. Cushingoid features, such as facial fullness, plethora, and dorsocervical and supraclavicular fat pad had resolved. Cortisol and ACTH levels were normalized. Mean serum CDC was 280 nmol/L (10 mcg/dL), and ACTH was 43 pg/mL (9 pmol/L). The 24-hour urine free cortisol (UFC) was 131 nmol/day (5 mcg/day), within the normal range. Interval CT of the thorax in March 2021 showed no further substantial reduction in size of the anterior mediastinal mass, measuring 2.1 cm × 0.8 cm × 1.6 cm (TS × LS × CC). The previously suspicious right lung nodules were no longer apparent.

His wife passed away, and he had stopped taking medications since August 2021. Two months later, he was found collapsed by a bystander when he went hiking alone in late October 2021. At the emergency department, he was disorientated with a Glasgow Coma Scale of 14/15, and blood pressure was 190/126 mmHg. He presented with more severe symptoms and electrolyte disturbances than at initial diagnosis. He had acute psychosis with visual and auditory hallucinations, severe hypokalemia of 1.5 mmol/L (mEq/L), extremely high serum cortisol of 5579 nmol/L (202 mcg/dL), and an ACTH level of 425 pg/mL (94 pmol/L). He was sent to the intensive care unit and was given etomidate infusion. Further complications were episodes of fast atrial fibrillation and methicillin-resistant *Staphylococcus aureus* (MRSA) pneumonia. After biochemical and clinical stabilization, he was managed with metyrapone 500 mg twice daily. He recovered, made good progress in rehabilitation, and was discharged 2 months later. Subsequent CT showed no interval change in size of mediastinal mass. He had a normal left ventricular ejection fraction by echocardiogram. There was no contradiction for surgery. However, patient declined surgery.

In the latest follow-up in August 2022, he was maintained on a metyrapone dose of 500 mg twice daily, while achieving a normal ACTH level of 23 pg/mL (5 pmol/L), a mean cortisol level of around 250 nmol/L (9 mcg/dL), and a normal 24-hour UFC.

## Discussion

In summary, this is a case of EAS due to presumably an ACTH-secreting thymic NET with suspicious lung metastasis in a 61-year-old gentleman, who presented with severe hypokalemia, metabolic alkalosis, hypertension, complicated with psychosis, vertebral collapse, and cardiac events. He had good biochemical and tumor response with metyrapone. Complete cure was not possible with metyrapone therapy alone, and a severe Cushing storm was triggered as a result of stopping medication. Although patient declined surgery, he remained independent in activities of daily living for more than 3 years since diagnosis while on metyrapone treatment.

In general, compared with Cushing disease (CD), EAS patients typically present with more severe and rapid onset of hypercortisolism symptoms, such as profound hypokalemia, metabolic alkalosis, resistant hypertension, hyperglycemia, hyperpigmentation, edema, and proximal myopathy. Long-term hypercortisolism features, such as significant weight gain, obesity, and violaceous striae are infrequent in EAS patients. Complications, including malignant hypertension, myocardial infection, thromboembolism, and osteoporotic vertebral fracture are also more acute in patients with EAS.

Thymic NET secreting ACTH is exceedingly rare, accounting for 5% to 10% of all cases of EAS [2]. In a recent systemic review of EAS due to thymic NET, those patients represented a younger population, under the age of 40, with a male preponderance [3]. The majority had aggressive hypercortisolism at presentation and had a median tumor size of around 5 cm. The reported mortality was 35.8%, and the median survival time was 38 months. Our patient was older (61 years), and the tumor size was smaller (3.6 cm in largest diameter) at diagnosis.

In view of the fulminant nature of EAS and mortality risk, a high clinical index of suspicion should be raised, especially in patients with intense hypercortisolism associated with ACTH concentration > 100 pg/mL (> 22 pmol/L) [3]. Radiological investigation must be prompt, while awaiting biochemical testing. Spiral thin-slice CT of the thoracic and abdominal regions, together with pituitary MRI, are the initial imaging of choice. Gallium-68 labeled somatostatin receptor PET/CT (^68^Ga-SSTR PET/CT) has been suggested to have superior performance to other imaging modalities in identifying EAS. Imaging with ^68^Ga-DOTATATE PET/CT has a sensitivity of up to 81.8% [4].

While bilateral inferior petrosal sinus sampling (BIPSS) is regarded as the gold standard to differentiate EAS from CD in ACTH-dependent CS, it is an invasive procedure. Noninvasive diagnostic strategies include corticotropin releasing hormone (CRH) test, desmopressin test, and high-dose dexamethasone suppression test. A study has shown that in patients with a negative pituitary MRI plus a positive CT scan of neck to pelvis, and negative responses to CRH and desmopressin tests, using the thresholds of a cortisol increase of > 17% and > 18, and an ACTH increase of > 37% and > 33% respectively, the negative predictive value was 100% for CD [5]. BIPSS was not performed for this patient, because of the absence of detectable pituitary tumor and a positive mediastinal mass seen on PET/CT scan. The high-dose dexamethasone suppression test has been controversial, and requires high-dose steroid administration, which may pose further risk to the patient. The CRH test was preferred in this case due to its good tolerability and short duration. The cortisol and ACTH responses to CRH stimulation in our patient were below threshold for CD. Together with the very high baseline ACTH level, negative pituitary MRI, and a ^18^FDG/^68^Ga-DOTATATE avid mediastinal tumor, the diagnosis of EAS was established.

In EAS, rapid control of excessive cortisol secretion is crucial. Metyrapone and ketoconazole are fast-acting oral steroidogenesis enzyme inhibitors that can reduce cortisol level within a few days and can lead to rapid improvement of hypertension and hypokalemia. The median dose of metyrapone used to control hypercortisolism in EAS patients was 1500 mg/day reported in a recent retrospective multicenter study [6]. Eucortisolism is defined as mean serum CDC value of 150 to 300 nmol/day (5-11 mcg/day) and a normal 24-hour UFC level. The dose of metyrapone required in our patient was approximately 1000 mg/day. Metyrapone causes a significant increase in 11-deoxycortisol, which may result in positive interference in cortisol immunoassay, such as the Beckman cortisol assay. In such case, 24-hour UFC ( by liquid chromatography–tandem mass spectrometry; LC-MS/MS) would be helpful.

Complete surgical resection remains the mainstay of curative treatment. Steroidogenesis inhibitors rarely suppress ectopic ACTH production and induce tumor regression, as the loss of negative feedback of cortisol induced may lead to increase in ACTH level. However, there have been case reports demonstrating ACTH normalization or partial suppression when given metyrapone or ketoconazole in patients with EAS [7–10]. Tumor regression was reported in a case of ectopic ACTH-producing lung tumor without surgery after 6 months of metyrapone treatment [7]. The exact mechanisms by which steroidogenesis inhibitors suppress ectopic ACTH production or tumor regression are unclear. It was postulated that there may be a direct effect of steroidogenesis inhibitors on ACTH secretion from the ectopic tumor, or that the tumor was regulated by high cortisol levels [10]. Based on current data, metyrapone is effective and safe for short- and long-term control of hypercortisolemia in CS. The most common side effect is mild gastrointestinal symptoms.

In the present case, both cortisol and ACTH levels were normalized with the use of metyrapone, and there was no tumor progression over 3 years. The initial suspected metastatic lung nodules were no longer apparent on subsequent CT scans. A follow-up ^68^Ga-DOTATATE PET/CT would be a better choice for evaluation. It was not performed due to financial burden to the patient.

In conclusion, although surgical resection of the tumor remains the best curative option, medical treatment with steroidogenesis inhibitors could be considered when the source of ACTH cannot be identified or when surgery is contraindicated or not feasible.

## Learning Points

- Ectopic Cushing syndrome presents with rapid onset of intense hypercortisolism features, which may include severe hypokalemia, uncontrolled hypertension, proximal myopathy, vertebral collapse, and psychiatric and cardiac complications
- Rapid control of hypercortisolemia and prompt imaging in search of tumor location are crucial in patients suspected of EAS
- Long-term metyrapone treatment has been demonstrated to be safe and effective, in terms of biochemical control and tumor stabilization, and it may serve as an alternative treatment in patients who are not able to undergo surgery

## Data Availability

Original data generated and analyzed during this study are included in this published article.

## Abbreviations

ACTH: adrenocorticotropic hormone
BIPSS: bilateral inferior petrosal sinus sampling
CC: cranial-caudal
CD: Cushing disease
CDC: cortisol day-curve
CRH: corticotropin releasing hormone
CS: Cushing syndrome
CT: computed tomography
EAS: ectopic ACTH syndrome
^18^FDG: fluorine-18-fluorodeoxyglucose
LS: length
MRI: magnetic resonance imaging
NET: neuroendocrine tumor
TS: transverse
